# Patient-Reported Outcome Measures of Breast Cancer Surgery: Evidence Review and Tool Adaptation

**DOI:** 10.7759/cureus.27800

**Published:** 2022-08-08

**Authors:** Sima Marzban, Samin Shokravi, Sadegh Abaei, Payam Fattahi, Maryam Karami, Faezeh Tajari

**Affiliations:** 1 Division of Research & Academic Affairs, Larkin Community Hospital, South Miami, USA; 2 Cancer Research Center, Shahid Beheshti University of Medical Sciences, Tehran, IRN; 3 School of Nursing & Midwifery, Shahid Beheshti University of Medical Sciences, Tehran, IRN

**Keywords:** breast cancer rehabilitation, breast conservation therapy, value based care, patient-centered outcomes research, breast cancer outcomes

## Abstract

The objective of this scoping review was to review survey instruments for Patient-Reported Outcome Measures (PROMs) and provide recommendations to construct a tool for PROMs specifically for breast cancer patients who have undergone surgery, to overcome the limitations of existing validated tools.

A total of 924 articles were screened. Nine articles were selected based on the eligibility criteria. We found that PROMs' data collection along with advancements in the treatment of breast cancer and the resultant improved clinical outcomes, there is a growing appreciation and focus on improving patients' quality of life (QoL). Previous studies have shown that the assessment of PROMs is linked to a positive effect on patients' symptoms of distress, quality of life, acceptance, and satisfaction. Several PROMs tools have been validated for use in cancer survivors. However, it is unclear whether existing tools are appropriate for use in breast cancer patients who have undergone surgical treatment. Hence, we conducted a scoping review.

Following a review of the current PROM related to breast cancer and the necessity to build specialized PROMs related to the outcomes of breast cancer surgery, we provide recommendations for the development of a comprehensive tool to overcome the limitations of existing PROMs tools.

## Introduction and background

Breast cancer is the most common cancer among women in 183 countries and the most common cause of cancer death in 115 countries. Statistics indicate that cancer incidence increased by 15% from 2005 to 2015 [1]. In the United States, the most common cancer in women in 2021 was breast cancer, with a prevalence of 30% and an estimated 90% five-year survival rate [2]. With increased survival rates, many patients experience physical and psychological sequelae of the disease in their daily activities, which reduce their Quality of Life (QoL) [3]. There are many functional aspects of breast surgery that can impact QoL: breast and shoulder/arm morbidity, both caused by breast tissue resection and axillary dissection [3-4]. Short-term (or postoperative) complications can lead to morbidity or even mortality. They are currently one of the main focuses of surgical research [5].

Short-term complications (especially within the first two weeks after surgery) include surgical site infection, skin necrosis at the reconstruction site, dehiscence, bleeding, hematoma, swelling of the upper extremities, lymphedema, numbness of upper extremities, decreased range of motion (ROM), pain, and dissatisfaction with body shape image. The highest prevalence of these complications occurs in the first month after surgery. Poor management of these difficulties in the postoperative follow-ups has been demonstrated to reduce QoL during the first year of surgery [3]. Furthermore, instantly distinguishing postoperative complications increase the treatment outcome, indicating the importance of tightly controlled observation of the patient during the first month of surgery. Using PROMs is an approach to the early identification of these side effects [6].

Traditionally, collecting the measured treatment outcome data is conducted by health care providers. Recently, value-based healthcare (VBHC) has been at the center of focus. Besides traditional oncologic and surgical outcomes, VBHC also measures the patient's desired outcome, referred to as patient-reported- outcome measures (PROMs) [4-5]. The International Consortium for Health Outcomes Measurement (ICHOM) has designed specific standard protocols for measuring outcomes. Due to the importance of breast cancer, it was one of the first concerns standardized to measure outcomes [4]. With the instruments designed by ICHOM, PROMs play a prominent role: about 75% of the outcome is measured by them, and the remaining 25% is measured clinically [7]. Now, PROMs have become the gold standard of measurement since the information obtained directly from the patient or authorized measurement tools reflects the main concerns and problems of the population [6]. PROMs allow patients to quantify their symptoms, functions, and expected therapy outcomes. In particular, PROMs in breast cancer surgery allow patients to evaluate the impact and effectiveness of the intervention [8-10].

This study is a narrative review of PROM instruments that measure treatment outcomes in cancer patients, especially those undergoing breast cancer surgery. based on our findings from our scoping review, we present recommendations for the development of a survey instrument to overcome the limitations of existing validated instruments.

## Review

Methods

This paper mainly discusses changes in QoL, particularly healthcare-related changes due to surgical intervention to treat breast cancer. PROMs are defined as a patient's evaluation and perception of their status in personal well-being (physical and psychological) or social relationships. Accordingly, this paper considers all the parameters altering the patient's perspective on QoL that can be affected by surgery. The study was conducted in two phases. First, the existing evidence was reviewed by a systematic search in several databases for original articles discussing QoL changes among breast cancer patients before and after surgery by collecting PROMs data. In the second phase, a questionnaire was developed to measure short-term outcomes in breast cancer patients who have undergone breast-conversing surgery.

Evidence Review

Study selection: The data collection was initiated by authors SA and PF. The databases were PubMed, Web of Science, Springer Link, and Google Scholar. The keywords were the MeSH terms: (PROM[Title/Abstract]) AND/OR (Patient-Reported outcome measure[Title/Abstract]) AND (Quality of Health Care[Title/Abstract])) OR (Quality of Life[Title/Abstract])) AND (breast neoplasms/psychology)) AND (breast neoplasms/surgery)) AND (breast neoplasms/therapy)] [1]. The terms were selected after performing preliminary research on the papers and consulting with a librarian specializing in health services research and systematic reviews. Studies describing translations of PROMs into languages other than English were investigated for references to original PROMs studies.

**Figure 1:**
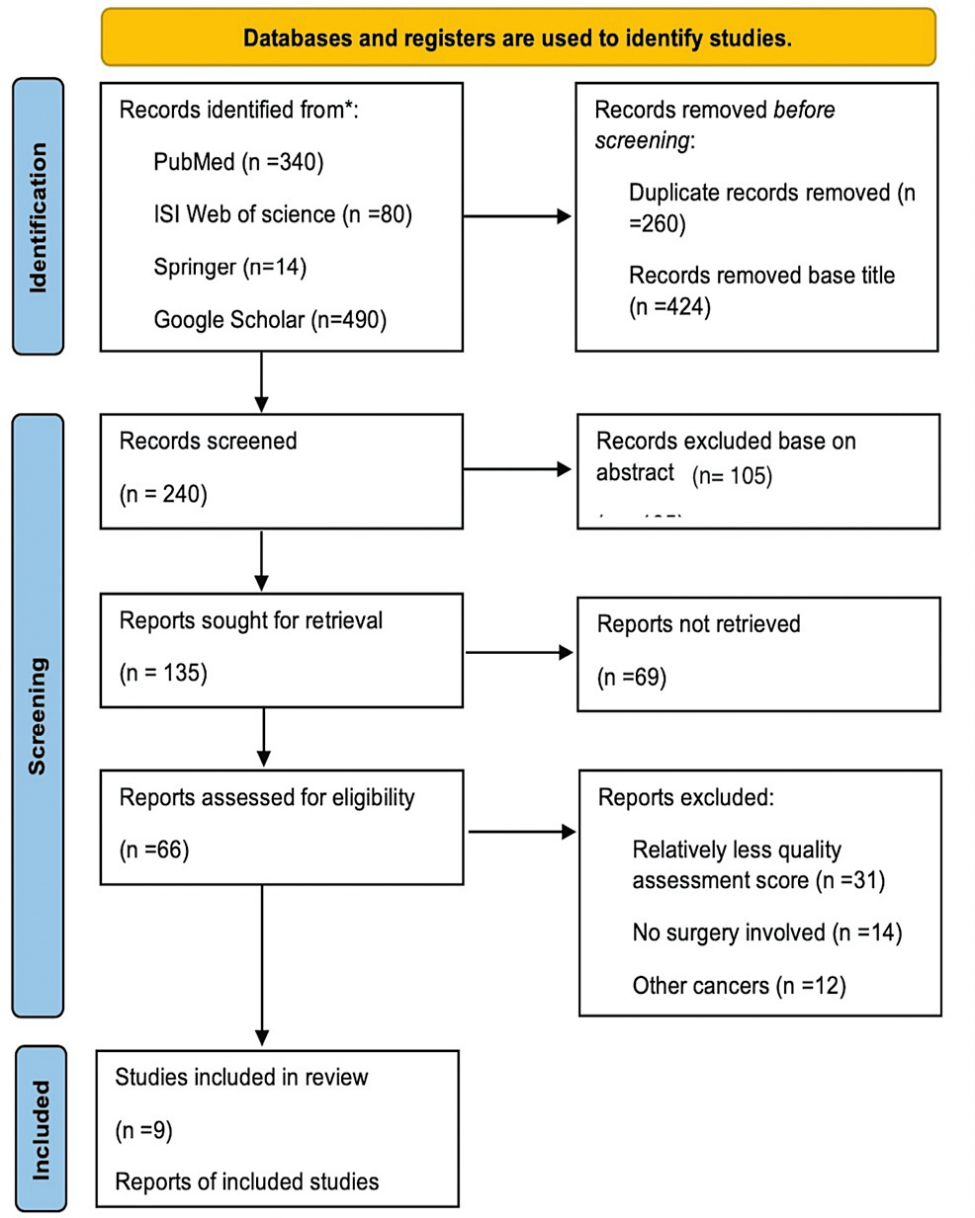
PRISMA 2020 flow diagram The PRISMA 2020 statement: an updated guideline for reporting systematic reviews [11] PRISMA: Preferred Reporting Items for Systematic Reviews and Meta-Analyses

Eligibility criteria: Author PF independently scored 10% of the abstracts on eligibility. Disagreements were resolved through discussion. A second reviewer was not required to select the full text, as the exclusion criteria were obvious. However, authors SA and PF independently evaluated the eligibility of nine articles. Furthermore, full texts studying the process of care rather than outcomes or primarily focusing on oncologic outcomes were excluded. Figure 1 shows the details of the literature selection process.

Data extraction of eligible studies:* *Author PF adjusted the initial draft of information extracted from eligible studies with items aligned with research objectives. SA reviewed this draft, which was revised after discussion. Each item was coded based on a four-attribute coding scheme as follows: It is recommended for use, it is not recommended for use, it may be used optionally, and a general statement about this item that does not include a recommendation. According to research objectives, the information obtained from the selected articles included the name of the first author, year of publication, the PROM instruments used, individual issues, community issues, and health care issues (see Appendices).

Appraisal methods: We used Preferred Reporting Items for Systematic Reviews and Meta-Analyses (PRISMA) guidelines for the scoping review and the PRISMA checklist can be found in the Appendices.

Recommendations for Constructing a Comprehensive Survey Tool

Future studies should consider making a standardized tool using nine components for a population of 350, using methodological considerations that we describe below.

Study population: The patient inclusion criteria in the questionnaire included definite breast cancer diagnosis, age (age > 18), consent to participate, and history of breast-conserving surgery (BCS). The exclusion criteria included psychiatric patients or conscious disorders, inability to comprehend or complete the scale due to weak language or intellectual capacity, and unwillingness to be the subject of research.

Distribution: Suggestion for the responder-to-item ratio varies from 5:1, 10:1, to 15:1 or 30:1 [12]. For example, for a ratio of 5:1 of 10 items of the questionnaire, there should be at least 50 respondents. Of course, the larger the number of respondents, the greater the validity of the questionnaire. Future studies should consider making a standardized tool using nine components for a population of 350 using the methodological considerations that we describe below.

Scale scores: For each element, patients responded using a five-point Likert scale to reflect how often they had experienced the problems over the past week. Initial values for each category ranged from 0 to 4. The responses were 0 = never, 1 = hardly, 2 = sometimes, 3 = often, and 4 = always.

*PROM Construction and Development* 

**Figure 2:**
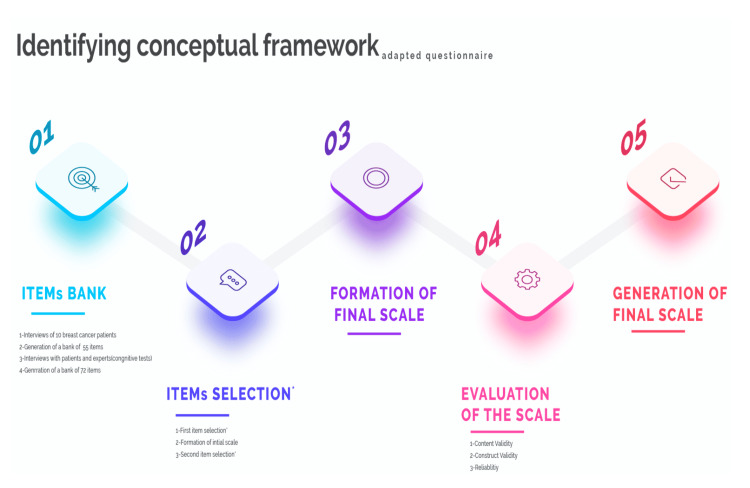
Conceptual framework of the adapted questionnaire

Below are the five steps of the developed PROMs tool designed for breast cancer patients who experienced breast-conserving surgery:

First, a comprehensive database of items was created. In an open interview with 10 breast cancer patients, the most critical problems they faced daily were listed. Then, a 55-item database was designed. A psychiatrist and methodologist were asked to check the items. Next, another database was developed that contained 72 questions. The items were selected in two phases. The main methods used in two item-selection processes were the classical test theory (CTT) and item response theory (IRT) [13]. The classical test theory is a set of related psychometric theories predicting psychological test results, such as the difficulty of the elements or the subject’s ability. The item response theory is a paradigm for designing, analyzing, and scoring tests, questionnaires, and similar instruments measuring abilities, attitudes, or other variables [13]. In the formation of the final scale, positively formulated items' notes were recoded as the original note plus one, whereas negatively formulated items' notes were recoded as five minus the original response. This recording resulted in a score range of 1 to 5 for each item, with a higher score indicating a more favorable PROM. The scale was evaluated based on these standards: initial validation, content validity, construct validity, and reliability of the designed PROM tool. See Figure 2.

Quality Assessment

The validity of the adapted questionnaire: The validity of a questionnaire is determined by evaluating whether the questionnaire measures what it should measure. In summary, the conclusions/outcomes based on questionnaire results (test scores) are valid. 

1. Initial validation - An experimental trial among respondents required initial validation. In this experimental trial, the final version of the questionnaire is administered to a large sample of survey respondents.

2. Content validity - Elements used to assess the validity of the content have the following features: questions were straightforward. The issues covered all areas of concern in breast surgery. The survey included essential questions about the breast surgery results. Finally, some of the issues were privacy-related.

3. Construct validity - Construct validity is the central concept in evaluating a questionnaire intended to measure a not directly observable concept (such as pain). To demonstrate the construct validity of our scale, it is possible to examine the extent to which patient responses on the new scale are correlated with confirmed instruments that also measure pain since they are measuring the same conceptual form.

Reliability of the Designed PROM

The adapted questionnaire's consistency was assessed using internal consistency, test‐retest reliability, and inter‐rater reliability.

Ethics

This project was introduced in the Research Ethics Committees of Cancer Research Center, Shahid Beheshti University of Medical Sciences, and the project was found to be in accordance with the ethical principles and national norms and standards for conducting medical research in Iran. The approval ID is IR.SBMU.CRC.REC.1400.047, and the certificate is available here: https://ethics.research.ac.ir/EthicsProposalViewEn.php?id=252978

Results

Search Diagram

Nine hundred twenty-four (924) articles, including abstracts and full texts, were screened for fulfilling all the above terms and satisfying the inclusion and exclusion criteria. Almost all were case series and therefore had level 4 evidence, while a few were meta-analyses. Two hundred sixty (260) were set aside, as they were found to be duplicates. Four hundred twenty-four (424) studies were excluded after the first screening for inclusion of other diseases were studied, surgical data were excluded, and the outcomes of oncological treatments were more promising, leaving 240 studies, out of which 66 full-text articles were found and analyzed. Upon further scrutiny, 57 were set aside because all of them used the same type of questionnaire and reported the same, comparable data. Only nine were finally selected for unequivocally studying and recommending relating the outcomes of the treatment of breast cancer from the patient's perspective (PROM). Table 1 summarizes the items from these nine questionnaires and compares them.

**Table 1:** Summary of results and adapted instruments These domains were divided into three groups based on their importance to the questionnaire's authors: ++, +, and -. In group ++, items from one domain accounted for more than 25% of the total content of the questionnaire. The items from the + group made up less than 25% of the total content of the questionnaire, and there were no items from the - group's domains in the questionnaire. BREAST-Q is a self-administered questionnaire. QLQ-BR23 is the breast cancer-specific quality of life questionnaire. QLQ-BR45 is the breast cancer-specific quality of life questionnaire. BIBCQ stands for body image after breast cancer questionnaire. FACT-B is the functional assessment of cancer therapy-breast. BCTOS refers to the breast cancer treatment outcome scale. SF-36 is the short form health survey questionnaire. PROMIS-29 is the patient-reported outcomes measurement information system. MBROS-S is the Michigan breast reconstruction outcome study.

Author name	Year of publication	Adapted instruments	Individual issues			Community issues			Health care issues		
			Physical aspects	Emotional	Self body-image	Social aspects	Sexual aspects	Domestic aspects	Surgery team	Nursing care	Equipment
AL Pusic et al. [14].	2009	BREAST-Q	+ (19/94) 20.2%	+ (7/94) 7.4%	+ (14/94) 14.8%	+ (10/94) 10.6%	+ (6/94) 6.3%	- (0%)	++(24/94) 25.5%	+ (14/94) 14.8%	- (0%)
A Montazeri et al. [15].	2000	QLQ-BR23	++ (23/23) 100%	- (0%)	- (0%)	- (0%)	- (0%)	- (0%)	- (0%)	- (0%)	- (0%)
V Bjelic-Radisic et al. [16].	2020	QLQ-BR45	++ (33/45)73.3%	+ (2/45) 4.4%	+ (2/45) 4.4%	+ (4/45) 8.8%	+ (4/45) 8.8%	- (0%)	- (0%)	- (0%)	- (0%)
Baxter et al. [17].	2006	BIBCQ	+ (6/50) 12%	+ (9/50) 18%	++ (29/50) 58%	+ (4/50) 8%	+ (2/50) 4%	- (0%)	- (0%)	- (0%)	- (0%)
Raymond Ng et al. [18].	2011	FACT-B	++ 15/37 40.5%	++ 13/37 35.1%	+ 3/37 8.1%	+ 5/37 13.5%	+ 1/37 2.7%	- (0%)	- (0%)	- (0%)	- (0%)
J Heil et al. [19].	2010	BCTOS	++ (8/12) 66.6%	- (0%)	++ (4/12) 33.3%	- (0%)	- (0%)	- (0%)	- (0%)	- (0%)	- (0%)
Elder et al. [20].	2005	SF-36	++ (20/36) 55.5%	++ (14/36) 38.8%	- (0%)	+ (2/36) 5.5%	- (0%)	- (0%)	- (0%)	- (0%)	- (0%)
Alla Sikorskii et al. [21].	2018	PROMIS-29	- (0%)	- (0%)	- (0%)	- (0%)	++ (37/37) 100%	- (0%)	- (0%)	- (0%)	- (0%)
Edwin et al. [22].	2000	MBROS-S	++ (26/48) 54.1%	+ (10/48) 20.8%	+ (7/48) 14.5%	- (0%)	+ (2/48) 4.1%	- (0%)	+(3/48) 6.2%	- (0%)	- (0%)

Developing an Adapted Version

Numerous questionnaires have been developed thus far to assess the efficacy of treatment modalities for breast cancer. In recent years, there has been a shift toward patient-centered questionnaires such as PROM. According to the data presented in Table 1, nine different questionnaires have been developed for use in this area; we have thoughtfully interpreted the items on each of these nine different questionnaires and the results obtained from them. Only three out of nine questionnaires (the body image after breast cancer questionnaire (BIBCQ), BREAST-Q, and MBROS-S questionnaires) have evaluated the outcomes of surgical interventions. In contrast, the majority of the remaining questionnaires have focused on the outcomes and side effects of oncological interventions (such as chemoradiotherapy). The MBROS-S and BIBCQ questionnaires have studied the majority of reconstructive, cosmetic, and self-body image after surgery; however, only the BREAST-Q questionnaire has studied both oncological and surgical outcomes in a more comprehensive manner. This questionnaire was used 18 months after the initial course of treatment to assess the patient's overall response to all of their treatments. Due to the high recall period of this questionnaire and the fact that it did not specifically examine the results of the surgery and evaluate all the treatments received by the patient as a whole, we decided to provide recommendations for designing a more comprehensive PROM tool that only examines all the results and complications of surgery from the patient's point of view. For this questionnaire, we considered a one-month recall period to examine surgery's problems and complications in greater detail. In addition, because the PROM questionnaire in our country, Iran, was not designed in accordance with our country's cultural context, we provided suggested items for use in regions with a particular cultural context.

The developed questionnaire includes three primary arms, nine domains, and 22 subdomains with 72 items. Each domain has a separate scoring, and there is no calculated total score. Items are scored from 0 to 100, with 0 being the lowest satisfaction and 100 being the highest satisfaction outcome.

The designed questionnaire has the following characteristics:

It takes 10 to 15 minutes to complete this questionnaire. It does not directly inquire about surgical complications. The questions have been designed so that the patient's desired outcome is deductible. Since the trends in breast surgeries are breast-conserving, covering a larger population of breast-operated patients with typically higher survival rates, we have decided to involve those who had undergone BCS in the study population. Also, a psychiatrist is invited to study the questionnaire and advocate suggestions to cover psychological issues exhaustively. The patients are asked to think aloud and express their understanding of the questions to ensure the eloquence of the pilot test questions.

**Figure 3:**
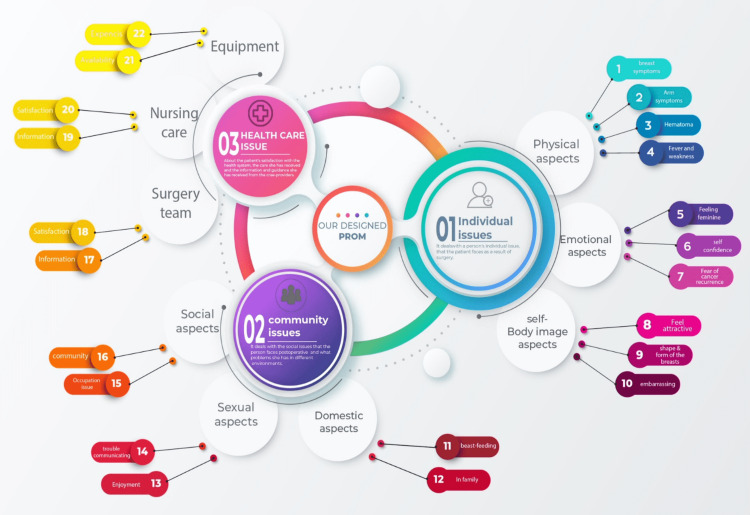
The domains of the designed patient-reported outcome measure

In this study, another questionnaire with 10 items was administered to assess the underlying problems related to the patient's preoperative surgery and enhance questionnaire reliability. To better evaluate the validity of the questionnaire, the score of each domain/theme was evaluated separately to compare results with similar items in other PROMs. The recall period was one week in the questionnaire. See Figure 3.

Discussion

Recently, in addition to traditional outcomes, including mortality and morbidity, patient outcomes and patient satisfaction have been given more weight in the assessment of treatment quality [4,23]. With subjective data collection and evaluation requiring unique methods and tools and the significance of evaluating such patient outcomes in promoting QoL, valid questionnaires have been developed to implement PROMs [24]. BIBCQ, Breast Q, BCTOS, FACT-B, SF-36, PROMIS, QLQ- BR45, and MBROS-S are PROM questionnaires designed to apply to breast cancer patients.

BREAST-Q

The BREAST-Q is a breast surgery-specific PROM developed in compliance with international guidelines for PROM instruments to assess patients’ satisfaction, HRQOL, and care experience developed in 2009. BREAST-Q version 1.0 was released in 2009 and version 2.0 in 2017. This shows minor changes to improve the language. Version 2.0 was tested in a much larger sample, and the scores obtained for both versions were similar. The Memorial Sloan Kettering Cancer Center and the University of British Columbia are the copyright holders of BREAST-Q^©^ [24].

The breast cancer module allows clinicians and researchers to tailor survey versions to their needs. The breast cancer cluster comprises mastectomy, reconstruction, breast reconstruction expectations, and breast-conserving therapy (BCT) [25].

Two main dimensions are used to build BREAST-Q clusters: health-related quality of life (HRQOL) and patient satisfaction. There are three subdomains for each site. Patient satisfaction includes satisfaction with the breasts, satisfaction with the outcome, and satisfaction with the care. QOL includes psychosocial, physical, and sexual well-being; patient satisfaction includes satisfaction with the breasts, satisfaction with the outcome, and satisfaction with the care [24].

EORTC (European Organization for Research and Treatment of Cancer) QLQ-BR45

The European Organization for Research and Treatment of Cancer (EORTC) designed PROM in 1986 as a questionnaire for patients' conception of their QoL. Then, in 1993, EORTC QLQ-C30 was published and soon became the central core of EROTC's quality of life assessments [26-27]. This questionnaire is the most commonly used PROM questionnaire in Europe and has been validated and earned global trust [27]. EORTC-QLQ-BR23 is a special breast cancer module designed to complete a general questionnaire. It has been studied internationally and is considered a credible and reliable tool for assessing the QoL associated with breast cancer [28]. QLQ-BR23 questions were mainly focused on patients' physical function; nevertheless, since 1996, with the multiple advances in the treatment of breast cancer in recent years, it was necessary to renew this questionnaire to pave the way for QLQ-BR45. QLQ-BR45 is the renewed version of BR23. It contains 22 new additions to the 23 items of BR23. New items mainly focus on the psychological condition and satisfaction of the patient. This questionnaire is in the IV phase of the investigation [29].

Unlike BREAST-Q, EORTC questionnaires are intended only for malignant tumors and have no role in evaluating breast surgeries, such as breast lumpectomy or reduction. See Table 2.

**Table 2:** Scale structure of EORTC QLQ-BR45 EORTC QLQ-BR45 is the European Organisation for Research and Treatment of Cancer quality of life questionnaire-breast 45 [16].

Scale structure of EORTC QLQ-BR45		
Functional Scale/Items	Symptom Scale/items	Target Therapy Scale
Body image	Systemic therapy side-effects	Endocrine therapy symptoms
Future perspective	Upset by hair loss	Skin mucositis symptoms
Sexual functioning	Arm symptoms	Endocrine sexual symptoms
Sexual enjoyment	Breast symptoms	
Breast satisfaction		

BIBCQ (Body Image After Breast Cancer Questionnaire)

Vamos introduced the body image after breast cancer questionnaire (BIBCQ) measure in 1993. It is based on the multidimensional aspects of chronic disease patients. Baxter et al. developed a version of this questionnaire in 2006 for the long-term evaluation of body image in patients with breast cancer [27]. The last version of BIBCQ contains 45 items, six optional items for lumpectomy patients (L items), and two optional items for mastectomy patients (M items) [17]. These items are classified into six domains as shown in Table 3:

**Table 3:** Body image after the breast cancer questionnaire (BIBCQ) scale Reliability and validity of the body image after breast cancer questionnaire [17].

BIBCQ Scale	
Vulnerability	items in this category sample feeling of susceptibility of the body to illness and cancer
Body stigma	items in this category sample the feeling of a need to keep the body hidden
Limitations	items in this category sample the feelings about competence and ability
Transparency	items in this category sample the concerns about the obviousness of cancer-related changes to one's appearance
Body Concerns	items in this category sample the concerns about the obviousness of cancer-related changes to one's appearance
Arm Concerns	items in this category sample the concerns about arm symptoms and appearance

On average, completing this questionnaire takes seven and a half minutes. Typically, 85% of patients complete it in under 10 minutes.

FACT-B (Functional Assessment of Cancer Therapy)

FACT-B is a 37-element instrument designed to measure five areas of HRQL in breast cancer patients: physical, social, emotional, and functional well-being, and a breast cancer subscale (BCS). Based on the 27 basic FACT-G elements, FACT-B was developed to emphasize patient values and brevity. Each element is assessed using a five-point Likert scale. The total FACT-B score is the sum of the scores of the five subscales, ranging from 0 to 144. A higher score denotes a better patient HRQoL [30].

In a relevant study published in the "Journal of Clinical Pathways," eight HRQoL instruments developed by different healthcare organizations were identified and evaluated. The FACT-B scored the highest after using the EMPRO (Evaluating the Measurement of Patient-Reported Outcomes) tool, with a cumulative score of 79.27. However, it was noted that other instruments might perform better in certain situations; for example, the EROTC BR23 and SF-36 scored higher than FACT-B on the validity attribute.

BCTOS-5 (Breast Cancer Treatment Outcome Scale)

This is one of the most well-organized and comprehensive PROMs. It is straightforward, research-friendly, comprehensible, and encompasses the essential aspects of post-treatment morbidity in terms of aesthetic and functional outcomes. It has been recently discovered that breast asymmetry, as judged by the BCTOS Cosmetic Scale, is associated with psychosocial functioning following breast-conserving surgery (BCS).

PROMIS-29 (Patient-Reported Outcomes Measurement Information System)

The National Institutes of Health (NIH) formed a cooperative group in 2004 to transform health measurement with the PROMIS. PROMIS-29 assesses each of the seven domains with four questions with an additional pain intensity numeric rating scale (NRS).

MBROS-S (Michigan Breast Reconstruction Outcome Study)

MBROS was started in September 1994 and continued until 2000. Its goal was to assess the long-term outcomes of implant, pedicle transverse rectus abdominis musculocutaneous (TRAM), and free TRAM breast reconstruction. Data on psychosocial, functional, and aesthetic issues are still under analysis. According to an analysis of psychosocial data, patients in all three surgical groups saw a significant improvement in general mental health, emotional well-being, and functional well-being after surgery [22].

ICHOM was founded in 2012 by Professor Michael Porter of Harvard Business School. It is an independent, not-for-profit organization firmly anchored in the healthcare-providing, scientific, and patient communities [21]. The consortium seeks to promote comprehensive standardized healthcare outcome measurements and align outcome measurement efforts globally. Its mission is to enable and facilitate value-based healthcare (VBHC) delivery worldwide and promote knowledge exchange about best practices across varying healthcare systems.

Despite the multiplicity of prom evaluation questionnaires, few questionnaires evaluate the outcome of patients who have experienced breast cancer surgery. BREAST-Q and QLQ- BR23 are the two existing PROMs introduced by ICHOM as a standard set for evaluating patients who have experienced breast cancer surgery. The BREAST-Q questionnaire evaluates long-term patient breast satisfaction. The QLQ-BR23 questionnaire primarily examines the arm and breast symptoms following adjuvant treatment, not specifically examining surgical outcomes. Given the lack of PROMs specifically addressing all aspects of short-term postoperative outcomes, a new questionnaire was developed in the present research. We suggest that surgical interventions be examined using the short term in order to examine the complications of surgery more precisely. On the other hand, by selecting a high recall time period, the patient may not remember some complications and problems resulting from the surgery.

Limitations and future directions

This scoping review is a bibliometric analysis of existing validated tools for PROM for breast cancer patients undergoing BCS. It is believed that this could potentially show repetitions, discrepancies, and areas where more investment is needed. Another limitation is that the pilot test of this questionnaire was limited to an Iranian community of cancer patients; thus, it should be measured in a larger statistical population in a native English-speaking community to ensure the required reliability and validity. To create a smaller list of issues, a larger community of patients should be interviewed. Therefore, in the next step, three phases are suggested to assess the quality of the new questionnaire, including conceptual framework formation (Phase I), item generation, preliminary scale formation, and pretesting (Phase II), and field-testing, scale construction, and psychometric evaluation (Phase III). In this study, we have dealt with Phases I and II, and we intend to implement this project in a metacentric Phase III.

## Conclusions

In recent years, following the improvement of breast cancer treatments, the overall survival has significantly increased. Currently, attempts are focused on improving the quality of life, although they should be more comprehensive and address more aspects of patients' personal lives. However, symptoms caused by various treatment techniques are still underestimated and require more serious attention. Pain, lymphedema, concern, and sexual function, particularly in young patients, and future outlooks are all issues that need further research to improve breast cancer patient's quality of life. Importantly, the terror of cancer should be broken, and patients should be motivated and convinced that cancer is not an end but merely a chapter in the long journal of life.

## Figures and Tables

**Figure 4:**
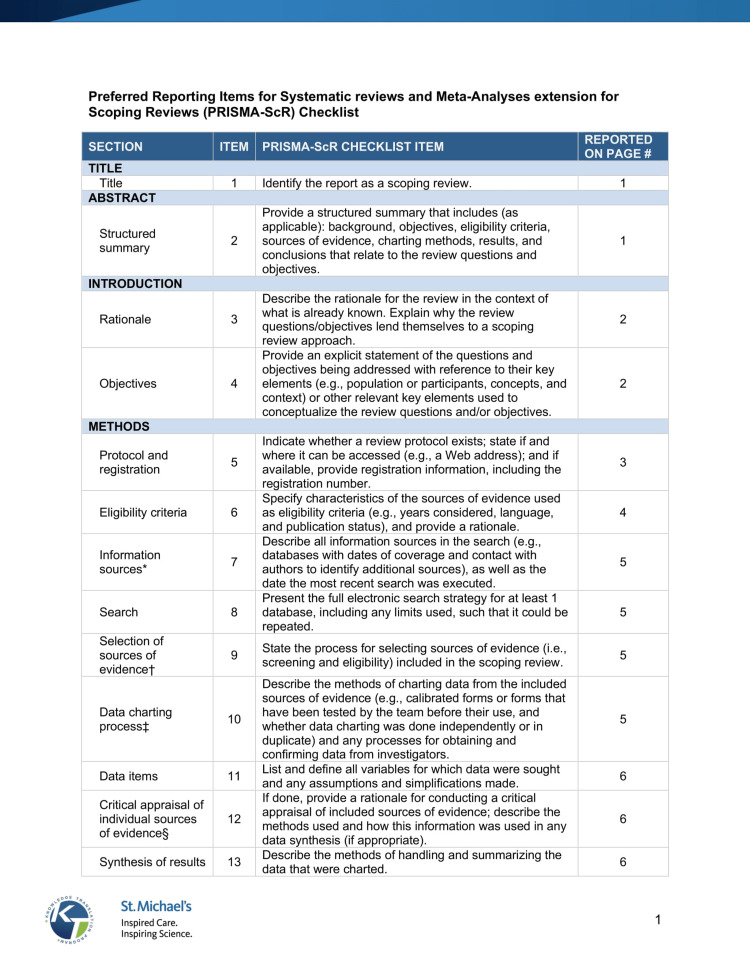
PRISMA Checklist for Scoping Reviews

**Figure 5:**
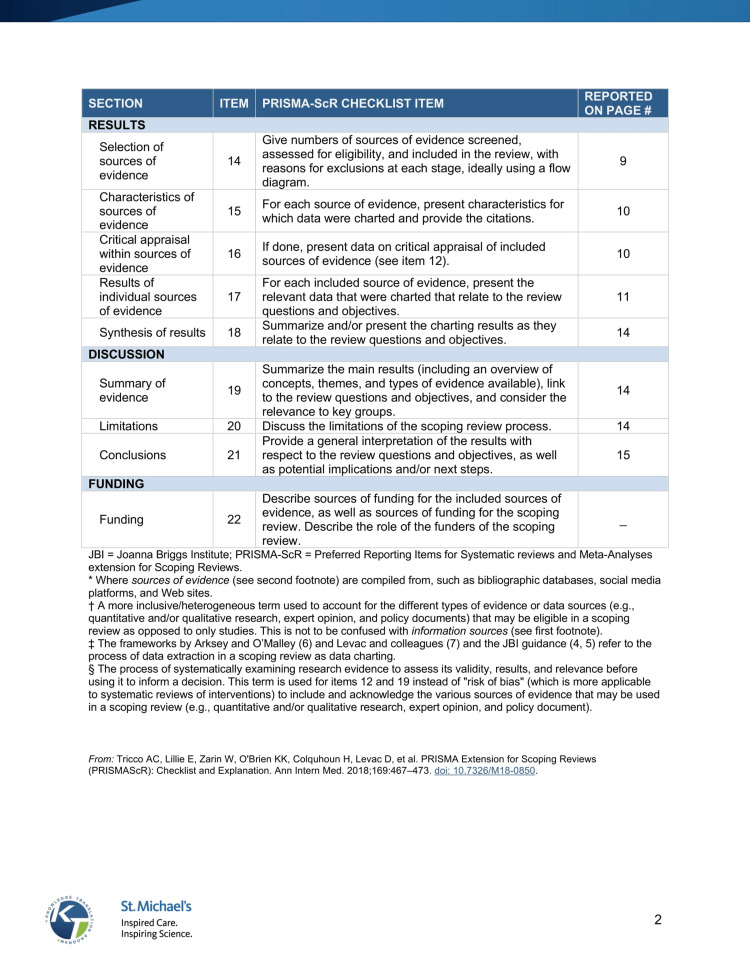
PRISMA Checklist for Scoping Reviews (Contd.)

**Figure 6:**
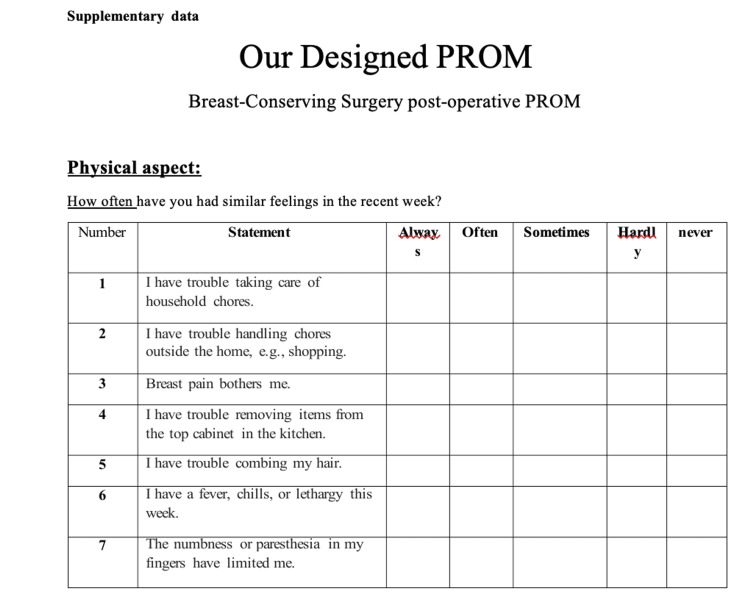
Physical Aspects

**Figure 7:**
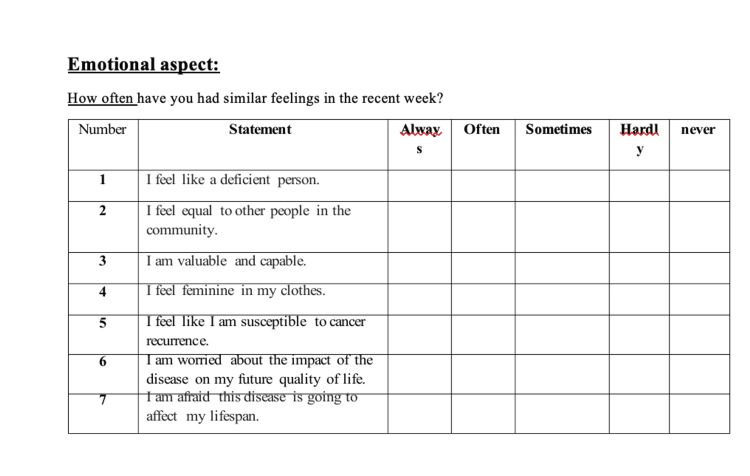
Emotional Aspects
